# Hand-held dynamometry in patients with haematological malignancies: Measurement error in the clinical assessment of knee extension strength

**DOI:** 10.1186/1471-2474-10-31

**Published:** 2009-03-09

**Authors:** Ruud H Knols, Geert Aufdemkampe, Eling D de Bruin, Daniel Uebelhart, Neil K Aaronson

**Affiliations:** 1 Department of Rheumatology and Institute of Physical Medicine, University Hospital Zurich, Switzerland; 2 University of Applied Sciences, Faculty of Health Care, Research Department of Lifestyle and Health, Utrecht, The Netherlands; 3 Institute of Human Movement Sciences and Sport, ETH, Zurich, Switzerland; 4 Division of Psychosocial Research and Epidemiology, The Netherlands Cancer Institute, Amsterdam, The Netherlands

## Abstract

**Background:**

Hand-held dynamometry is a portable and inexpensive method to quantify muscle strength. To determine if muscle strength has changed, an examiner must know what part of the difference between a patient's pre-treatment and post-treatment measurements is attributable to real change, and what part is due to measurement error. This study aimed to determine the relative and absolute reliability of intra and inter-observer strength measurements with a hand-held dynamometer (HHD).

**Methods:**

Two observers performed maximum voluntary peak torque measurements (MVPT) for isometric knee extension in 24 patients with haematological malignancies. For each patient, the measurements were carried out on the same day. The main outcome measures were the intraclass correlation coefficient (ICC ± 95%CI), the standard error of measurement (SEM), the smallest detectable difference (SDD), the relative values as % of the grand mean of the SEM and SDD, and the limits of agreement for the intra- and inter-observer '3 repetition average' and the 'highest value of 3 MVPT' knee extension strength measures.

**Results:**

The intra-observer ICCs were 0.94 for the average of 3 MVPT (95%CI: 0.86–0.97) and 0.86 for the highest value of 3 MVPT (95%CI: 0.71–0.94). The ICCs for the inter-observer measurements were 0.89 for the average of 3 MVPT (95%CI: 0.75–0.95) and 0.77 for the highest value of 3 MVPT (95%CI: 0.54–0.90). The SEMs for the intra-observer measurements were 6.22 Nm (3.98% of the grand mean (GM) and 9.83 Nm (5.88% of GM). For the inter-observer measurements, the SEMs were 9.65 Nm (6.65% of GM) and 11.41 Nm (6.73% of GM). The SDDs for the generated parameters varied from 17.23 Nm (11.04% of GM) to 27.26 Nm (17.09% of GM) for intra-observer measurements, and 26.76 Nm (16.77% of GM) to 31.62 Nm (18.66% of GM) for inter-observer measurements, with similar results for the limits of agreement.

**Conclusion:**

The results indicate that there is acceptable relative reliability for evaluating knee strength with a HHD, while the measurement error observed was modest. The HHD may be useful in detecting changes in knee extension strength at the individual patient level.

## Background

Intensive medical treatment regimens can significantly improve survival in patients with haematological malignancies [1-3]. The cancer therapy itself, including chemotherapy or radiotherapy, damages healthy cells throughout the body, resulting in side-effects including nausea, emesis, decreased nutritional intake and anaemia. Higher fatigue levels that are associated with decreased levels of activity and lengthened bed rest contribute to muscular catabolism and atrophy [4]. As a result, functional limitations and muscle weakness may persist even well beyond the period of active treatment [5-7].

Patients with haematological malignancies may benefit from physical exercise programs in terms of maintenance or even improvement in physical activity levels [7], fitness levels [8,9], and muscular strength [5,10,11]. Assessment of muscle strength is an important part of the management of cancer patients, particularly in determining the response to a muscular strength training program [12-14]. It is thus important to be able to accurately quantify the muscle strength of patients who are recovering from intensive medical treatment.

Muscular strength can be assessed both in research settings and in clinical practice settings by means of isokinetic and hand-held dynamometers (HHD). One of the advantages of using isokinetic dynamometers in patients with chronic diseases is the ability to assess muscle strength dynamically through a range of movements at various velocities, which may more accurately reflect functional performance [15,16]. However, isokinetic strength testing protocols may be too time consuming in typical clinical settings, and the size of the equipment can also be problematic (i.e., lack of portability). Clinically, HHD represents a simple, portable and relatively inexpensive alternative to isokinetic machines for assessing muscle strength [16]. Moreover, hand-held dynamometers provide quantification of muscle strength, and are more sensitive to change in muscle strength than simple manual muscle tests [16,17].

Evidence of the validity of HHD has been provided in several studies, including a comparison of HHD with isokinetic strength measurements to assess lower limb strength in the elderly (r = 0.91) [18], a comparison of HHD and manual muscle testing (r = 0.77) [17], and of HHD and the Timed-Up-and-Go-test (r = 0.64 to -0.94). [19] Nollet et al. also provided evidence for the validity of a HHD in lower strength ranges in patients with post-polio syndrome [20].

To be clinically meaningful, however, the muscle strength assessment procedure must be reliable enough to evaluate outcomes of a therapeutic intervention [21]. Reliability can be reported in *relative *or *absolute *terms [21]. Relative reliability statistics indicate the degree of association between 2 or more measures (e.g., intraclass correlation coefficients or ICCs), [22] but they do not provide clinical guidance for assessing real changes at an individual patient level [23,24]. The relative reliability of hand-held dynamometers for knee extension has been examined in numerous populations. ICCs' of 0.75 or higher have been reported in studies of healthy young and elderly adults [25,26], community-dwelling elderly fallers [19,27], people with acquired brain injury [28], elderly after hip fracture and elective hip and knee arthroplasty [29,30], adults with cerebral palsy [31], and patients with chronic obstructive pulmonary disease (COPD) [16].

*Absolute *reliability reflects the magnitude of the differences between two measures [32]. Examples of these statistics are the standard error of measurement (SEM), the corresponding 95% confidence interval, the smallest detectable difference (SDD), and the limits of agreement (LA). To be clinically useful, an assessment with an HHD must have only a small amount of measurement error in detecting real change over time.[33] A retest difference in a patient with a value smaller than the SEM is likely to be the result of 'measurement noise' and is unlikely to be detected reliably in practice; a difference greater than the SDD is likely to be a real difference with 95% certainty [21]. The absolute reliability of HHD has been reported by several authors [16,26,27,31,33,34]. However, measures of reliability are specific to the populations and testing procedures used. This implies that the findings of previous studies may not be applicable to patients with haematological malignancies. Disease- and treatment-related symptoms, including de-conditioning, muscle weakness, and fatigue may affect not only the reliability, but also the safety of performing HHD [16,22]. Therefore, the investigation of the measurement error of an HHD in patients with haematological malignancies is warranted.

In daily physiotherapy or rehabilitation practice, strength measurements for the same patient are often performed by several examiners. However, the measurement error associated with the assessment of strength by one observer (intra-observer reliability) may be different than that associated with the assessment of strength by several observers (inter-observer reliability)[35]. For this reason, it is important to determine both the intra- and inter-observer reliability of the measurements obtained with a HHD. This study aimed to determine the relative and absolute reliability (measurement error) of intra and inter-observer strength measurements with a HHD in a sample of patients with haematological malignancies.

## Methods

### Selection criteria

The study sample included patients with a diagnosis of haematological cancer who had completed treatment with high-dose chemotherapy in the Departments of Oncology and Haematology of the University Hospital Zurich. Patients were excluded if they were experiencing the direct side-effects of high-dose chemotherapy (e.g. fever, haemoglobin level < 10 g/dl, emesis, dyspnoea, ≤ 36 of 52 points on the Functional Assessment for Cancer Therapy-Anemia (FACT-An) scale [36,37]), or had gait abnormalities, known impairment of the lower limbs, severe graft versus host disease (GVHD) except for grade I not requiring treatment, painful joints, instable osteolyses of the vertebrae, chronic low back pain, lesions of the central or peripheral nervous system, uncontrolled cardiovascular disease, thyroid disease, or diabetes.

Forty-nine patients were initially invited to participate in the study. Five of these patients (10.2%) were subsequently excluded due to low haemoglobin values and/or severe fatigue, 2 patients (4%) were excluded due to knee pain at the time of measurement, and 12 patients (24%) were not interested in participating. Of the remaining 30 patients, 14 had leukaemia treated with induction chemotherapy following peripheral blood stem-cell transplantation, 11 non-Hodgkin lymphoma treated with high-dose chemotherapy alone (n = 10) or high-dose chemotherapy following autologous stem cell transplantation (n = 1) and 4 multiple myeloma/plasmacytoma treated with high-dose chemotherapy alone (n = 1) or high-dose chemotherapy following 2 cycles of autologous stem-cell transplantation (n = 3). All participating patients were in a physically stable condition and provided written informed consent. The ethics committee of the Canton of Zurich approved the study.

### Descriptive measurements

Blood values (haemoglobin in g/dl) were determined at the time of an outpatient visit to the hospital. Self-reported fatigue was measured with the German-language version of the FACT-An scale. [37] The FACT-An scale includes 13 items relating to both the symptoms and consequences of cancer fatigue, and is highly reliable [37]. Haemoglobin values and self-reported fatigue were assessed because both of these variables (i.e. low haemoglobin levels and high fatigue levels) can have adverse effects on physical performance over time [38]. The patient's height was assessed to the nearest 0.5 cm with a wall fixed tape measure. Weight was assessed to the nearest 0.5 kg with a weighting machine, SECA^©^, Model 791.

### Isometric muscle strength assessment

The maximum voluntary push torque (MVPT) was assessed with the CompuFet HHD. The CompuFet is a portable *force *evaluation and testing system (weight 0.45 KG), designed by Hoggan Health Industries Inc. (USA). The HHD sets a high or low threshold for the minimal *force *with which to start. The high threshold recording of the test data begins at 13.6 Newton. The display shows peak force read-outs in 4.4 Newton increments. The CompuFet HHD has a test-range from 3.6 to 440 Newton [39].

### Standardization of the measurement protocol

**Figure 1:**
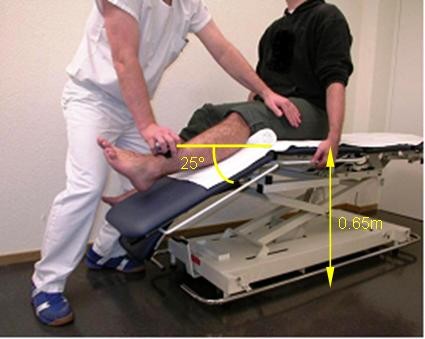
**Standardization of measurements**. Performance of a maximum voluntary peak torque assessment at a knee angle of 25° of flexion. Standard body position of the patient and the device are shown. The patient stabilized the trunk by grasping the table.

The MVPT for knee extension was tested at a knee angle of 25 degrees [33]. An angle of 25 degrees was selected to correspond to the knee angle at which the force production is of crucial importance in walking, as has been shown in biomechanical analyses of this activity [33,40,41]. Patients were positioned sitting upright, with no back support, and with the hips in 90 degrees flexion. The patient stabilized the trunk by grasping the table. The thigh of the patient was stabilized by the examiner's hand. Thus, the examiner assured that sufficient counterforce was produced by the thigh, so that the lower limb could not pivot down during the break test with the knee near full extension. In this way, the examiner could ensure that the knee extension was really "broken". The joint angles were defined according to the Academy of Orthopaedic Surgeons (AAOS) system [42]. The HHD was positioned perpendicular to the tibia, at 80% of the shank length (between the marks at the lower edge of the 'lateral epicondylus' and the lower edge of the 'lateral malleolus'), distal to the knee. The knee joint centre and the 80% shank length were marked with a dot on the patients' skin. The position of the patient, the examiner, and the HHD were standardized (Figure 1).

The test was performed as a 'break test'. The break technique requires the examiner to overpower a maximal effort by the patient, thereby producing a measurement of eccentric muscle strength [43]. The break technique produces higher values than the 'make technique'. The make technique requires the patient to exert a maximal isometric contraction while the examiner holds the dynamometer in a fixed position. Both the break and the make method (ICCs for both methods are 0.90 or higher) produce strength measurements that have excellent reliability, although the 'break' technique produces higher values [44]. The patient's forced exertion was standardized according to 'Caldwell', with a build-up phase of 2 seconds, and steady maximal force exertion over 3 seconds, after which the examiner breaks through the forced exertion of the patient [33,45]. The patient was encouraged by means of standardized, verbal instructions during the tests. The break test requires sufficient force from the examiner [46]. For this study, MVPT for isometric extension strength measurements was expressed in Newton-meter (Nm). A concave interchangeable patch attachment for curved surfaces [39] was used to avoid pain at the tibia during the assessment. The average of 3 peak torque measurements and the highest value of 3 peak torque measurements were used as outcomes. The knee extension score was estimated from the torque signal, multiplied by the measured lever arm between the HHD device and the knee joint.

### Measurement procedure

The test procedure started with a familiarization session of three knee extension repetitions of the dominant limb [21], which was defined as the preferred limb for kicking. The rest interval between the test repetitions was 30 seconds. The reliability study started with one examiner (intra-observer reliability) performing two measurement sessions of three repetitions each. Subsequently, the second examiner (inter-observer reliability) performed a third measurement session of three repetitions. Thus, a total of nine repetitions on the dominant limb were performed by each patient. The measurement sessions, including the training session, were separated by 60 minutes. After one hour, no real change in muscle strength in patients is expected, so any observed differences were expected to be due to measurement error. In addition, the break-interval is long enough to avoid muscular fatigue effects [33].

The reliability of the knee extension measurements was evaluated at the Institute of Physical Medicine at the University Hospital Zurich by two examiners, both female students (examiner 1: 80 Kg, 1.64 m, and examiner 2: 53 Kg, 1.62 m) from the Institute of Human Movement Sciences and Sport of the ETH, Zurich. As neither examiner had previous experience with manual muscle testing, they underwent training sessions to learn the requisite manual muscle testing skills. They practised the manual muscle testing skills on fellow students, and on 3 patients with haematological malignancies. The students practised the muscle strength measurements during 8 sessions for 1.5 hours, totalling approximately 12 hours. During this training they were supervised by a senior physical therapist (RHK) with experience in manual muscle testing.

### Statistical Analyses

Normality of the data was tested with the Kolmogorov-Smirnov test [47]. A two-way mixed model (ICC_3.1 _and ICC_3.3_) and a two-way random effect model (ICC_2.1 _and ICC_2.3_) were used for the intra, and inter-observer reliability estimation, respectively [48]. An ICC > 0.75 was defined as acceptable reliability. The SEM was calculated from the average known standard deviation (SD) and the relative reliability coefficient (ICC) of the measurement used for our sample: SEM = SD(√ 1-ICC) [49,50]. The corresponding 95% confidence interval (95%CI), in which the true score (drawn from the normally distributed population) is expected to fall, was ± 1.96 × SEM [33,51,52]. The broader the limits of the 95% confidence interval, the less confident the estimation of the true score and, as a consequence, the less confident the detection of real change due to intervention [50]. This knowledge about the standard error of the measurement is necessary before one can say that a change has occurred [50,53].

Moreover, when analyzing a difference between two consecutive observations, one must consider the standard error of the observed score for both the first (SEM_(first measurement session)_) and the second (SEM_(second measurement session)_) observations. The SDD is defined as the measure of statistically significant change between two independently obtained measurements. Given a probability value of α = 0.05 as indication for statistical significance, the SDD is estimated as 1.96 × √(SEM_(first strength assessment)_^2 ^+ SEM_(second strength assessment)_^2^) [24]. Assuming that the standard error of the measurement of the observed score of the first and second observations are equal, the SDD is 1.96 × √2 × SEM. For a statistically significant change between two separate observations to be detected, this change must be at least the SDD of the measurement procedure [49]. The SEM and SDD's were expressed as absolute values and in relative values as % of the grand mean.

The limits of agreement (LA) were calculated as the difference against the mean plot (LA = mean + 1.96 × SD) as proposed by Bland and Altman [54]. The Bland and Altman plots graphically display between measurement differences, thus allowing direct insight into the variability of the measurement under study [55].

A repeated measures ANOVA was carried out to test for learning effects within the three MVPT strength measurements [56]. The differences between means of the intra-observer and inter-observer measurements (p < 0.05) were calculated with a paired t-test [22]. Sociodemographic differences between patients included and excluded from the study were calculated by means of a Student's t-test [57]. All statistical analyses were performed using SPSS^® ^15 for Windows (SPSS, Inc.).

## Results

The HHD-assessments were tolerated by all 30 patients. Of these 30 patients, 6 (1 woman and 5 men) were excluded from the analyses because they did not perform the knee extension measurements according to the standardized procedures and because they exceeded the torque limit of 218 Nm. These 6 patients were significantly younger, taller and heavier (p < 0.05), than the 24 patients included in the analysis (Table 1).

**Table 1:** Descriptive characteristics

Demographic and medical measures	Patients with haematological malignancies (n = 24; Included for analyses)	Patients with haematological malignancies (n = 6; Excluded for analyses)	p-value
Mean Age in years (sd)	50.1 (14.6)	39.5 (8.9)	0.042
Mean Height in cm (sd)	170.1 (7.7)	183.5 (8.1)	0.001
Mean Weight in kg (sd)	72.2 (11.7)	87.5 (9.3)	0.007
Mean BMI in kg/m^2 ^(sd)	24.8 (3.9)	26.1 (1.4)	0.359
Mean 80% shank-length in cm (sd)	32.3 (2.2)	36.3 (1.50)	0.000
Mean time (days) since medical treatment (sd)	149.0 (70.2)	148.7 (69.2)	0.991
FACT-An (13 fatigue items) (Sd)	40.5 (7.0)	37.5 (11.7)	0.426
Haemoglobin (g/dl) (Sd)	12.4 (1.7)	12.9 (2.3)	0.545

Mean values for included and excluded patients with hematological malignancies. Differences were calculated by an unpaired t-test.

For the remaining 24 patients, all results of the muscle strength measurements and the difference in muscle strength measurements between intra-session 1, intra-session 2, and the inter-session were normally distributed. The relative reliability of the HHD, including the ICCs and the 95%CIs, was acceptable, ranging from 0.77 to 0.94, for the intra-observer and inter-observer measurement sessions, respectively (Tables 2 and 3).

**Table 2:** Relative and absolute reliability of the average of 3 MVPT measurements

Intraclass correlation coefficient model	ICC (± 95%CI)	SEM (SEM as % of grand mean)	SDD (SDD as % of grand mean)
Intra observer ICC model 3.3	0.94 (0.86 – 0.97)	6.22 (3.98%)	17.23 (11.04%)
Inter observer ICC model 2.3	0.89 (0.75 – 0.95)	9.65 (6.05%)	26.76 (16.77%)

The Intraclass correlation coefficient (ICC), the standard error of measurement (SEM), the smallest detectable difference (SDD) and the % of the grand mean for the intra- and inter-observer average MVPT value of knee extension strength measurements in patients with haematological malignancies are presented.

**Table 3:** Relative and absolute reliability of the highest value of 3 MVPT measurements

Intraclass correlation coefficient model	ICC (± 95%CI)	SEM (SEM as % of grand mean)	SDD (SDD as % of grand mean)
Intra observer ICC model 3.1	0.86 (0.71 – 0.94)	9.83 (5.88%)	27.26 (17.09%)
Inter observer ICC model 2.1	0.77 (0.54 – 0.90)	11.41 (6.73%)	31.62 (18.66%)

The Intraclass correlation coefficient (ICC), the standard error of measurement (SEM), the smallest detectable difference (SDD) and the % of the grand mean for the intra- and inter-observer highest MVPT value of knee extension strength measurements in patients with haematological malignancies are presented.

**Figure 2:**
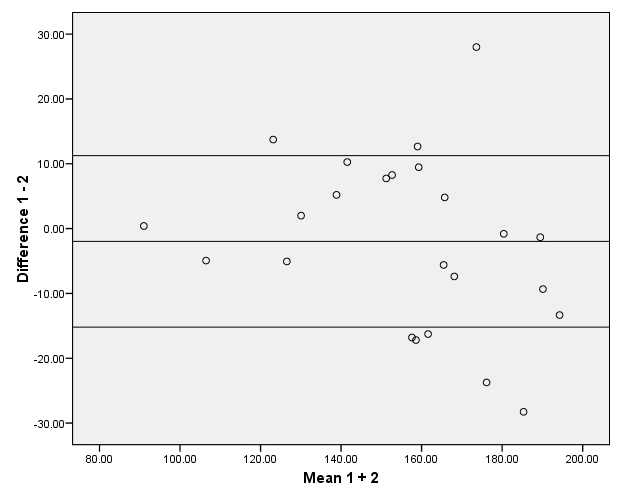
**Distribution from Bland and Altman; average of 3 MVPT values for intra-observer measurements**. Eight data points are outside the ± 1.96 standard deviation boundaries. The ± 1.96 standard deviation boundaries represent approximately 15.19 Nm below and 11.25 Nm above the mean, which is -1.97 Nm.

**Figure 3:**
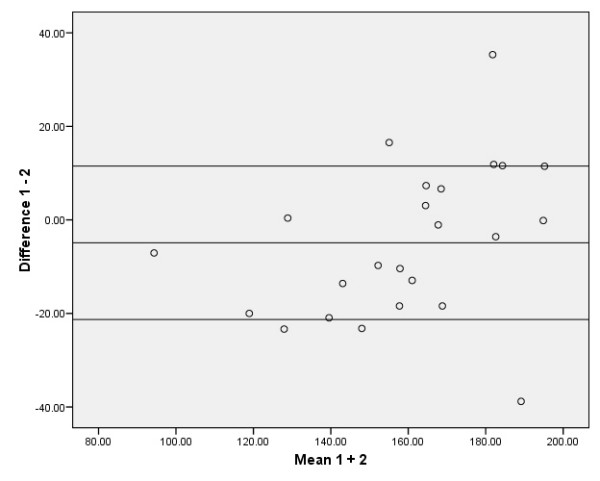
**Distribution from Bland and Altman; average of 3 MVPT values for inter-observer measurements**. Five data points are outside the ± 1.96 standard deviation boundaries. The ± 1.96 standard deviation boundaries represent approximately 21.29 Nm below and 11.51 Nm above the mean, which is -4.89 Nm.

**Figure 4:**
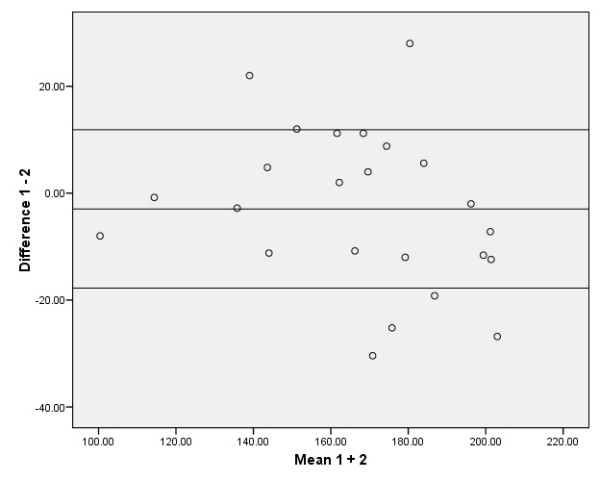
**Distribution from Bland and Altman; single MVPT values for intra-observer measurements**. Six data points are outside the ± 1.96 standard deviation boundaries. The ± 1.96 standard deviation boundaries represent approximately 17.77 Nm below and 11.87 Nm above the mean, which is -2.95 Nm.

**Figure 5:**
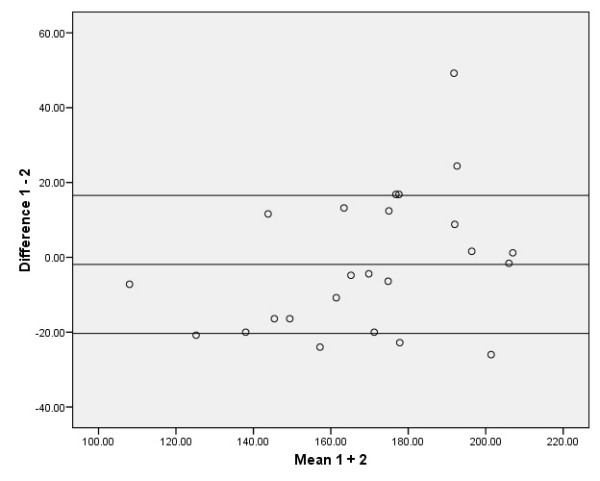
**Distribution from Bland and Altman; single MVPT values for inter-observer measurements**. Five data points are outside the ± 1.96 standard deviation boundaries. The ± 1.96 standard deviation boundaries represent approximately 20.36 Nm below and 16.56 Nm above the mean, which is -1.9 Nm.

The absolute reliability of the SEM, the SDD, and the relative values as % of the grand mean of the SEM and SDD are presented in Tables 2 and 3. The 95% limits of agreement according to the method of Bland and Altman are presented in Figures 2, 3, 4, 5. The ANOVA for repeated measures yielded no significant changes (p > 0.05) between the three MVPT strength measurements, indicating that there were no learning effects from the first to the third measurements for the intra- and inter-tester observers. There were no significant differences in muscle strength between the intra- and inter-observer sessions for the average of 3 MVPT measurements (Tables 4 + 5) or for the highest value of 3 MVPT measurements (Tables 6 + 7).

**Table 4:** Average MVPT values for intra-observer measurements

Examination	Grand mean (sd)	Stand. error. mean	p-value
Intra-Observer 1	155.5 (25)	5.1	0.508
Intra-Observer 2	157.1 (29.1)	5.9	

Mean (sd), and standard error of the mean for the average value of 3 MVPT intra-observer strength measurements.

**Table 5:** Average MVPT values for inter-observer measurements

Examination	Grand mean (sd)	Stand. error. mean	p-value
Inter-Observer 1	165.6 (26.9)	5.4	0.340
Inter-Observer 2	168.5 (30.0)	6.1	

Mean (sd), and standard error of the mean for the average value of 3 MVPT inter-observer strength measurements.

**Table 6:** Highest MVPT values for intra-observer measurements

Examination	Grand Mean (sd)	Stand. Error. mean	p-value
Intra-Observer 1	157.1 (29.1)	5.9	0.310
Intra-Observer 2	160.4 (23)	4.7	

Mean (sd), and standard error of the mean for the highest value for 3 MVPT intra-observer strength measurements.

**Table 7:** Highest MVPT values for inter-observer measurements

Examination	Grand Mean (sd)	Stand. Error. mean	p-value
Inter-Observer 1	168.5 (30)	6.1	0.619
Inter-Observer 2	170.4 (23.8)	4.9	

Mean (sd), and standard error of the mean for the highest value for 3 MVPT inter-observer strength measurements.

## Discussion

This study evaluated the relative and absolute reliability of a strength assessment protocol using an HHD among a sample of haematological cancer patients recovering from high-dose treatment. We used the ICC (with accompanying 95%CI) to estimate relative reliability. Relative reliability is highly dependent on the variability observed in the patient sample, and relates to the ability to classify patients' strength measurements in the same rank. Thus, relative reliability is most relevant for assessing instruments that are to be used for discriminative purposes [23]. Guyatt et al.[24] demonstrated that discriminative instruments require a high level of relative reliability. That is, the measurement error should be small in comparison to the variability between the observers. In other words, if the difference between the observers is large, a certain amount of measurement error is acceptable [23,24].

However, if the aim is to measure change in health status, which is often the case in clinical practice, absolute reliability is more relevant [23,24]. Absolute reliability describes the agreement between repeated measurements and is concerned with measurement error [23,24]. For an evaluative instrument, it is not the variability between the observers that is of primary concern, but rather measurement error [23,24]. The measurement error should be smaller than the changes that the observer wishes to detect [23,58]. We calculated the SEM, the SDD and the limits of agreement to estimate absolute reliability.

To be of practical use, the results should be interpreted as follows: the intra-observation of the average of 3 MVPT 'knee strength' assessments provided acceptable relative reliability (ICC_3.3 _= 0.94). The reliability of this parameter is affected by the variance statistic of the assessments from 'intra session 1', which was 644.65 Nm (calculated as the square of the standard deviation [25.39 Nm]), and the assessment from intra session 2', which was 847.39 Nm (sd 29.11 Nm) (see the distribution from Bland and Altman in Figure 2). When taking the measurement error into account, an SDD equal to or greater than 17.23 Nm between two measurements should be used as the threshold for a true clinical change in knee extension. The results of the other examination models in this study: the Inter-observer reliability for the average of 3 MVPT measurements (ICC_2.3_), the intra-observer reliability for the highest value of 3 MVPT measurements (ICC_3.1_), and the inter-observer reliability for the highest value of 3 MVPT measurements (ICC_2.1_), should be interpreted in the same way (see Tables 2 and 3, and Figures 3, 4, 5). Thus, when evaluating knee strength measurements (e.g. after a muscle strength program), it is recommended to use the 3-repetition average strength measurement by one or more examiners.

We performed intra- and inter-observer re-test measurements on the same day. However, no learning effect was found in the present study between the first and the third strength measurement. This is probably due to the familiarization session [21]. Although the highest value is probably a more valid measurement for assessing muscle strength [59] (even though it is less reliable), the average of three MVPT strength measurements can be used in determining whether a result is a real change or is within the range of measurement error.

The protocol used for assessing isometric knee strength in this study had acceptable re-test reliability, as evidenced by ICCs equal to or greater than 0.75. The ICCs in the current study are similar to test-retest reliability coefficients reported in other, related studies [16,19,25-31].

The measurement error of HHD for knee extension strength in haematological patients can be compared to that observed in other studies. In a study in orthopaedic knee patients, the intra-observer assessment of the SDD was 21.5 Nm for the single value, and 13.8 Nm for the average value. For inter-observer assessment, the SDD was 28.2 Nm for the single value and 18.7 Nm for the average value [33]. However, one should keep in mind that the authors used the 'make' method to assess knee extension strength.

To compare our absolute reliability results for knee extension strength with those observed in COPD patients [16], we estimated the SEM from their results. The SEM was estimated from the ICC and the total variance, using the formula SEM = Sd × (√1-ICC) [48]. A SDD (= SEM × 1.96 × √2) of approximately 49 Nm from knee extension was calculated from their study results (ICC .87, Sd 14.5 Nm, strength value originally expressed in Kg, converted to Nm and corrected to an average lever arm of 34 cm, which was the average 80% shank length of the included and excluded participants in our study, (see table 2) [16]. An important difference from our measurement protocol was that the measurements in this study were performed with a knee angle in 90 degrees of flexion.

From the study of Taylor et al. [31] among patients with cerebral palsy, we were able to calculate a SDD of approximately 43 Nm (ICC .81, Sd 10.7, strength value originally expressed in Kg, converted to N and corrected to an average lever arm of 34 cm for Nm).

Excellent SEMs in knee arthroplasty patients were described by Gagnon et al. The average SEM from 3 trials was 1.84 Nm (SDD 5.10 Nm) [34]. However, in this latter study, a chair-fixed device was used, and therefore was not fully comparable with the results of hand held dynamometry. In contrast to chair fixed dynamometry, the reliability of strength measurements in HHD is influenced by the experience of the examiners, the amount of strength that examiners are able to resist, and the standardization of measurements [33].

Currently, there is no criterion for the SDD of hand held dynamometry. Therefore, the SDD in knee extension strength was compared to studies that obtained quadriceps strength measures after a resistive strength exercise program. A relatively small improvement of 18 Nm (95%CI 7–30 Nm, GM 144, Sd 45 Nm) was found in patients with COPD [60]. Conversely, we estimated a mean change of 29.92 Nm (CI95% 24 Nm to 35 Nm) from the results of a study of breast cancer patients [12]. Although muscle strength in this study was assessed with an eight repetition maximum, which is not fully comparable to HHD, the findings indicated that cancer patients may benefit from muscle strength training during chemotherapy treatment. Taken together, if obtained by the same observer, the SDD threshold of 17 Nm (see table 2) that corresponds to the average of 3 MVPT strength measurements, will probably be surpassed.

For the average inter-examiner MVPT measurements with the HHD, it is questionable if the threshold of 26 Nm (see table 2) will be surpassed in all haematological patients after a strength resistive training program. However, this is probably the case only in patients who recover steadily from the side effects of the medical treatment, and who are good responders to resistive strength training.

Several limitations of the current study should be mentioned. First, the resultant moment at the knee joint and the moment by the dynamometer are different. When measuring isometric strength, one should keep in mind that the differences between the measured and the resultant joint moments might influence the estimation of muscle torque parameters. Although the test protocol can be standardized to a reasonable degree, the deformation at the soft tissue of the leg, especially at the thigh, where the muscle mass is considerable, plays an important role in changing the alignment of the HHD axis of rotation, and the axis of the knee joint [61]. Therefore, future studies need to examine the 'real' joint angles of hand-held dynamometry measurements.

Second, the measurements in this study were performed by female examiners without prior experience in muscle strength assessment with HHD. This may have influenced the upper boundary of the muscle strength assessments. Knee extension strength measurements performed by stronger examiners with experience in hand-held dynamometry may result in measurement values that are higher than 218 Nm. Moreover, the use of an isokinetic dynamometer has been recommended if the muscle strength of the patients exceeds the strength of the examiners [21]. In several studies, isokinetic dynamometers yielded reproducible measurements with low measurement error [21,61-63]. However, isokinetic dynamometers also have several disadvantages. They require a good deal of space, and are costly, hampering their widespread use in clinical settings. The reliability of a HHD measurement may depend on the strength and the body mass of the examiner. The female examiners in this study were of varying weight. Examiner 2 achieved the highest (mean) MVPT measurements.

Third, the point in time at which the assessments took place varied considerably (see Table 1), and therefore some patients may have had the possibility to recover more from the side-effects of high-dose chemotherapy than others. This may have influenced the inter-subject variability, which in turn increases relative reliability (ICCs). However, this inter-subject variability does not effect absolute reliability (SEM, SDD). It is also possible that the patients in our study were healthier than other haematological cancer patients at the same stage of recovery. The primary reason that 12 patients did not participate was that they felt too fatigued or too weak to do so.

Fourth: although we could not detect a learning effect between the MVPT measurements, one should keep in mind that the results of this reliability study are based on an intra-day reliability assessment. A more complete picture of the reliability would require a between-day reliability study to allow the corresponding variations to affect (or not) the measures. Learning effects for strength measurements can potentially be of more concern for between-day than for within-day measurements [64,65]. In addition, if truly maximal exertions of muscle strength are desired, visual feedback should be employed during the measurements [66]. A factor that may also influence the reliability of strength measurements is the circadian rhythm. A time-of-day effect for leg and back strength measurements was reported in one study in which maximum strength values increased consistently during daytime [67]. Gauthier et al. [68] reported similar findings for elbow flexion torque and body temperature, which varied concomitantly during the day. One should keep in mind that circadian rhythm disruption is hypothesized as a mechanism underlying fatigue in cancer patients [69]. Fatigue is one of the most prevalent symptoms that cancer patients experience and it has a considerable effect on physical performance [70]. Therefore, fatigue may also influence the reliability of the measurements in cancer patients.

Fifth, at the end-phase of the training period, the upper limit for the examiners torque was fixed at 218 Nm, because the weakest examiner was able to break through the knee extension movement of the 3 pre-test patients at 218 Nm, but not higher. Thus, only haematological cancer patients with knee extension measurements lower than this value were included in the analysis.

Finally, this study had a relatively small sample size. Although the sample size was adequate for studies of this nature [71], a larger study might narrow the confidence intervals around the reliability coefficients (without necessarily affecting the reliability estimates themselves).

### Clinical implications for the use of a HHD in patients with haematological malignancies

In this reliability study both participating assessors were students of the Institute of Human Movement Sciences and Sport. They underwent training sessions to learn the requisite manual muscle testing skills during 8 sessions of 1.5 hours each. The data for the average intra-examiner MVPT measurements in 24 patients with hematological malignancies yielded acceptable results for relative (ICC 0.94) and absolute reliability (SDD 17 Nm).

The conflicting finding on inter-examiner reliability, where the experience of the assessing examiners seemingly plays an important role, has important clinical implications. If more than one examiner is to evaluate the muscle strength of a haematological patient, then it is important that all examiners concerned apply the tests reliably and consistently. If this can not be achieved, then the resulting data will be of little use in a clinical setting. Clinicians specialized in the treatment of chronic diseases, and with comparable levels of practical experience with an HHD can, however, use the average MVPT value for intra-examiner measurements in their everyday practice with confidence. The HHD may be used in patients with haematological malignancies who have recovered from the direct side-effects of their medical treatment and who are in a stable physical condition to: 1) compare muscle strength with normative reference values (e.g. for discriminative purpose); or 2) evaluate the effect of a resistive exercise training in an individual patient (e.g. measure change in health status over time).

## Conclusion

The results of this study indicate that there is acceptable relative reliability for evaluating knee strength with an HHD, while the observed measurement error is modest. The HHD may be useful in detecting changes in knee extension strength at the individual patient level.

## Abbreviations

COPD: Chronic obstructive pulmonary disease; FACT-An: Functional Assessment of Cancer Therapy-Anaemia; GVHD: Graft versus host disease; HHD: Hand-held dynamometer; ICC: Intraclass correlation coefficient; LA: Limits of agreement; MVPT: Maximum voluntary peak torque; Nm: Newton-meter; SDD: Smallest detectable difference; SEM: Standard error of measurement; 95%CI: 95% Confidence interval.

## Competing interests

The authors declare that they have no competing interests.

## Authors' contributions

RHK is the guarantor of the study. He designed the study and was the main writer of the manuscript. GA and EDB designed and wrote the study, and critically revised the study for its content. DU initiated and monitored the study. NKA supervised and critically revised the study for its content. All authors read and approved the final manuscript.

## Pre-publication history

The pre-publication history for this paper can be accessed here:


